# Metabolomics-based elucidation of the chemical basis under-lying seasonal variations in sensory quality of Goldsands Black Tea

**DOI:** 10.3389/fpls.2026.1888622

**Published:** 2026-07-01

**Authors:** Jialin Chen, Binghong Liu, Jiaxin Li, Yixin Chen, Yide Zhou, Hui Meng, Xindong Tan, Peng Zheng, Binmei Sun, Hongbo Zhao, Shaoqun Liu

**Affiliations:** 1 Country College of Horticulture, South China Agricultural University, Guangzhou, China; 2 Goldsands Tea Research Institute, South China Agricultural University, Guangzhou, China

**Keywords:** flavor chemistry, Goldsands Tea, seasonal variation, sensory quality, untargeted metabolomics, volatile metabolomics

## Abstract

Goldsands Black Tea (GBT), a premium black tea from northern Guangdong, exhibits seasonal sensory variations with unclear chemical basis. This study integrated sensory evaluation, volatile and untargeted metabolomics to characterize the flavor profiles of spring, summer, and autumn GBTs. Spring tea achieved the highest sensory score, characterized by sweet aroma and fresh, mellow taste; autumn tea presented complex floral-fruity notes; summer tea showed pronounced astringency. Volatile metabolomics identified 66 compounds, with nine showing significant seasonal differences. Five compounds—geraniol, methyl salicylate, linalool, (*E*)-*β*-ionone, and *β*-myrcene—were key aroma contributors. 82 core differential compounds were identified from 9894 ion features detected by non-volatile metabolomics. Amino acids accumulated predominantly in spring tea, positively correlating with freshness/sweetness, while flavonoids accumulated in summer/autumn teas, positively correlating with astringency. Pathway analysis identified phenylpropanoid metabolism, amino acid metabolism, and flavonoid biosynthesis as key regulatory pathways. This study provides initial insights into the chemical basis of seasonal quality variations and offers guidance for optimizing production.

## 1 Introduction

Black tea is the most widely consumed tea category globally, valued for its distinctive organoleptic properties (Li et al., 2013; Liu et al., 2023). The northern Guangdong region of Shaoguan, encompassing Yingde City and Renhua County, possesses a long history of tea cultivation. The unique terroir of this area—characterized by Danxia landforms, karst topography, and a subtropical monsoon climate—is recognized for fostering exceptional tea germplasm diversity (Kong et al., 2025). As a UNESCO World Natural Heritage site, the Danxia landscape has yielded the discovery of several novel *Camellia* species in recent years, underscoring its significance as a biodiversity hotspot (Lin et al., 2024; Wang et al., 2026). Goldsands Black Tea (GBT) is a representative black tea variety derived from the ancient wild tea tree populations native to this Danxia landscape. It exhibits a characteristic aroma profile featuring cinnamon and rose notes, attributed to its specific genetic background and regional adaptation.

The formation of tea quality is influenced by multiple factors, including cultivar genetics, harvesting season, and processing techniques. Among these, the harvesting season is recognized as a key environmental determinant shaping tea sensory characteristic (Ye et al., 2022; Chen et al., 2026). Prior research has demonstrated that climatic variations across spring, summer, and autumn significantly alter the accumulation patterns of secondary metabolites in tea plants, thereby leading to discernible differentiation in the flavor quality of finished teas (Gong et al., 2020; Huang et al., 2022; Chen et al., 2025b). For example, studies on Huhong Congou black tea revealed that sweet and umami amino acids are significantly more abundant in spring teas, whereas flavonol glycosides accumulate preferentially in summer teas (Jiang et al., 2021). Similarly, investigations into Rucheng Baimao black tea confirmed that key aroma components, such as geraniol and methyl salicylate, display distinct seasonal accumulation patterns, with spring harvests exhibiting optimal aroma quality (Zhu et al., 2025).

Despite this body of knowledge, specific research on the seasonal sensory variation of GBT remains absent. The chemical basis underlying the seasonal flavor characteristics of this emerging rare variety is not yet elucidated, which currently limits the scientific foundation required for its standardized production and targeted quality regulation.

Therefore, the present study aimed to test the hypothesis that differential environmental factors across spring, summer, and autumn regulate primary and secondary metabolic pathways in GBT, leading to distinct accumulation profiles of volatile and non-volatile metabolites that subsequently shape the seasonal sensory quality. To address this hypothesis, we integrated sensory evaluation with two complementary metabolomic approaches: headspace solid-phase microextraction gas chromatography–mass spectrometry (HS-SPME-GC-MS) for volatile profiling and ultra-performance liquid chromatography-tandem mass spectrometry (UPLC-MS/MS) for non-volatile profiling. Multivariate statistical analysis was employed to screen for key differential metabolites and to construct association networks linking metabolite profiles to specific sensory attributes.

## 2 Materials and methods

### 2.1 The sample of tea

**Figure 1:**
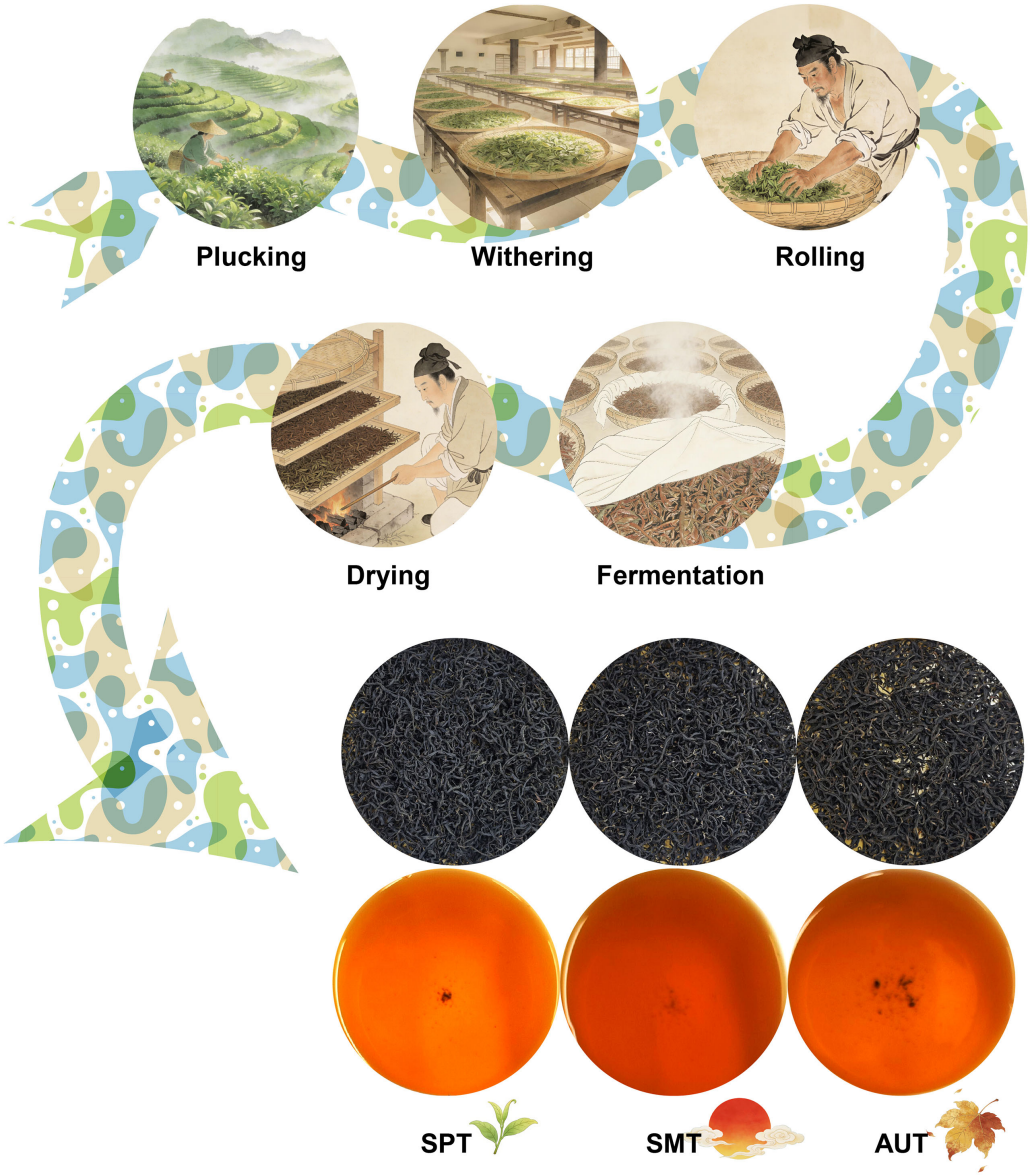
Processing workflow results of spring, summer, and autumn GBT. Spring tea (SPT), Summer tea (SMT), Autumn tea (AUT).

All tea samples used in this study were derived from Goldsands Tea (*Camellia sinensis* var. Goldsands), with fresh leaves harvested from the Goldsands Tea Plantation Yard in Shaoguan City, Guangdong Province, China (25°3’N, 113°47’E). The leaves were plucked according to the standard of “one bud with two leaves” and subsequently processed into black tea following a standardized manufacturing protocol (**Figure 1**): fresh leaves (one bud and two leaves) → slot withering (20–25 °C, 12–20 h) → rolling (combined light and heavy pressure, total duration 60–150 min) → fermentation (26–28 °C, 6–10 h) → drying (100–120 °C, 60–180 min). Based on the harvest season, the samples were categorized into three groups: Spring Tea (SPT), harvested in April 2025; Summer Tea (SMT), harvested in July 2025; and Autumn Tea (AUT), harvested in October 2025. The tea leaves were harvested from multiple tea plants within the same plantation and pooled to prepare a representative sample.

### 2.2 Chemicals and materials

The alkane standard solution (C9–C21) for calculating linear retention indices (RIs) came from TanMo Quality Testing Technology Co., Ltd. (Beijing, China). The internal standard solution was prepared with dichloromethane before use (Chen et al., 2025a). Ultrapure water was produced with a Barnstead GenPure Pro system (Thermo Fisher Scientific, Waltham, MA, USA). Methanol, acetonitrile, isopropanol, formic acid, and acetic acid were from Fisher Scientific (Waltham, MA, USA). Ammonium formate, ammonium acetate, and ammonia solution were from Aladdin Bio-chemical Technology Co., Ltd. (Shanghai, China).

### 2.3 Analysis of volatile compound

#### 2.3.1 Headspace solid-phase microextraction

The extraction of volatile compounds was carried out by HS-SPME, based on the procedure reported by Chen et al (Chen et al., 2025b). Briefly, 2.0 ± 0.02 g of homogenized tea powder was placed into a 40 mL headspace vial. After adding 5 mL of saturated NaCl solution, 10 μL of internal standard (IS) solution was introduced into the sample matrix. The IS solution was prepared by two-step dilution of ethyl decanoate (density 0.864 g/mL): first, 1 μL of ethyl decanoate was dissolved in 999 μL of dichloromethane to yield Solution A (864 μg/mL); subsequently, 10 μL of Solution A was diluted with 990 μL of dichloromethane to obtain Solution B (8.64 μg/mL). The addition of 10 μL of Solution B thus delivered a consistent amount of IS (~86.4 ng ethyl decanoate) to each sample. The vial was immediately sealed with a PTFE-lined aluminium cap. The mixture was equilibrated in a metal bath at 80 °C for 15 min, after which a divinylbenzene/carboxen/polydimethylsiloxane (DVB/CAR/PDMS) fibre (50/30 μm film thickness, 2 cm length; Supelco, Darmstadt, Germany) was exposed to the headspace at 80 °C for 40 min. The fibre was then retracted and transferred to the GC injection port where thermal desorption was performed at 250 °C for 3 min in splitless mode. All samples were analysed in triplicate (six replicates for the pooled quality control sample); the IS was used throughout for signal normalisation.

#### 2.3.2 Gas chromatography–mass spectrometry analysis conditions

Analyses were performed on an Agilent 1890B gas chromatograph coupled with a 5977A mass spectrometer (Agilent, Santa Clara, CA, USA). Separation was achieved on an HP-5MS capillary column (30 m × 0.25 mm i.d., 0.25 μm film thickness). Helium was used as carrier gas at a constant flow rate of 1.0 mL/min in splitless mode. The oven temperature program was set as follows: initial temperature 50 °C held for 1 min, increased to 220 °C at 5 °C/min, and held for 5 min. The mass spectrometer was operated in electron ionization (EI) mode at 70 eV, with the ion source temperature at 230 °C, and the quadrupole at 150 °C. Mass spectra were recorded over a scan range of m/z 30–400 with a solvent delay of 4 min.

#### 2.3.3 Identification and quantification of volatile compounds

Volatile compounds were identified by comparing their mass spectra with those in the NIST14 library and by matching their linear RIs with reference values. RI values were calculated using a series of n-alkanes (C_9_–C_21_) analyzed under identical chromatographic conditions, according to the following Equation 1:

(1) $$\begin{array}{c} \;RI=100\text{n}+100\times \frac{\text{RT}(\text{x})\;-\text{ RT}(\text{n})}{\text{RT}(\text{n}+1)\;-\text{ RT}(\text{n})}\; \end{array}$$

where RT(x), RT(n), and RT(n+1) are the retention times (min) of the target compound, the n-alkane with carbon number n, and the n-alkane with carbon number n+1, respectively. A compound was considered positively identified when its RI deviated by less than 15 units from the NIST14 database and its spectral similarity exceeded 90%.

Quantification of volatiles was performed using the internal standard method. The concentration of each compound was calculated as follows (Equation 2):

(2) $$\begin{array}{c} {\;}_{\text{C>i}}\frac{{=}_{\text{S>i}}}{{\text{S}}_{\text{is}}}\times \frac{{\text{m}}_{\text{is}}}{\text{m}} \end{array}$$

where C_i_ is the concentration of compound i (μg/kg), S_i_ is the peak area of compound i, S_is_ is the peak area of the internal standard, m is the sample mass (g), and m_is_ is the mass of internal standard added (ng).

#### 2.3.4 Calculation of relative odor activity values

The rOAV for each volatile compound was calculated by dividing its concentration by its odor threshold in water, which is widely used to evaluate the contribution of individual compounds to the overall tea aroma. An rOAV ≥ 1 indicates that the compound contributes significantly to the aroma profile, whereas an rOAV < 1 suggests it is not detectable by the human nose (Lu et al., 2023). The calculation is expressed as (Equation 3):

(3) $$\begin{array}{c} \text{r>O>A>V}\frac{\;}{{{=}_{\text{C>i}}}_{\text{T>i}}} \end{array}$$

where C_i_ is the concentration of compound i (μg/kg) and T_i_ is its odor threshold (μg/kg).

### 2.4 LC-MS-based untargeted metabolomics analysis

#### 2.4.1 Sample preparation

Tea samples were thawed on ice and inspected for integrity. Each sample (50 ± 2 mg) was accurately weighed into a 2.0 mL microcentrifuge tube, and 500 μL of ice-cold 80% methanol (LC-MS grade) was added. The mixture was homogenised at 45 Hz for 180 s and then incubated at −20 °C for 1 h to complete protein precipitation and metabolite extraction. After centrifugation at 20, 000 × g for 15 min at 4 °C, 250 μL of the supernatant was transferred to a clean 1.5 mL tube and re-centrifuged under the same conditions. Finally, 200 μL of the cleared supernatant was transferred to an LC-MS autosampler vial. All steps were performed on wet ice to minimise metabolite degradation. The fixed sample weight (50 mg) and constant extraction volume (500 μL) ensured consistent matrix loading across all samples. Statistical data normalisation was subsequently performed using Probabilistic Quotient Normalization (PQN). Five biological replicates were prepared for each sample.

#### 2.4.2 UPLC-HRMS conditions

Chromatographic separation was performed on an ACQUITY UPLC system (Waters, Milford, MA, USA) equipped with an HSS T3 column (100 mm × 2.1 mm, 1.8 μm, Waters). The mobile phase consisted of (A) water containing 5 mM ammonium acetate and 5 mM acetic acid, and (B) acetonitrile. The gradient elution program was as follows: 0–1 min, 1% B; 1–9 min, 1%–99% B; 9–9.1 min, 99%–1% B; 9.1–12 min, 1% B. The flow rate was 0.35 mL/min, injection volume was 2 μL, and column temperature was maintained at 40 °C.

Mass spectrometric analysis was performed on a Q Exactive HF mass spectrometer (Thermo Fisher Scientific, Waltham, MA, USA) equipped with a heated electrospray ionization (HESI) source, operating in both positive and negative ion modes. The ion source parameters were set as follows: ion transfer tube temperature, 350 °C; spray voltage, +3.8 kV (positive mode) and 4 kV (negative mode); sheath gas flow rate, 50 Arb; auxiliary gas flow rate, 15 Arb; sweep gas flow rate, 0 Arb. Data acquisition was performed in Full MS/dd-MS² (data-dependent acquisition) mode. Full scan spectra were acquired over a mass range of *m/z* 70–1050 at a resolution of 70, 000, with an automatic gain control (AGC) target of 3E6 and a maximum injection time of 100 ms. For dd-MS², the top five most abundant ions exceeding an intensity threshold of 100, 000 were selected for fragmentation at a resolution of 17, 500, with an AGC target of 1E5 and a maximum injection time of 50 ms. Normalized collision energies were set at 20, 40, and 60 eV. Dynamic exclusion was set to 6 s.

#### 2.4.3 Data processing and metabolite identification

Raw LC-MS data were converted to mzML format using MSConvert (ProteoWizard) and processed with XCMS in R for peak detection, retention-time alignment, and peak grouping. Isotopes and adducts were annotated using MetaboAnnotation and CAMERA. The resulting three-dimensional data matrix (retention time–*m/z* pairs, sample names, ion intensities) was subjected to rigorous quality filtering: (i) features with >80% missing values across biological samples or >50% missing values across QC samples were removed; (ii) remaining missing values were imputed using the K-nearest neighbour (KNN) method; (iii) only features with a relative standard deviation (RSD) ≤ 30% in the pooled QC samples were retained for statistical analysis, thereby ensuring that only robust and reproducible signals were considered. Signal intensity drift was assessed using the QC samples and, if necessary, corrected by locally estimated scatterplot smoothing (LOESS) regression. Procedural blanks (80% methanol processed identically to samples) were analysed alongside the study samples; features whose mean intensity in blanks exceeded 20% of the mean intensity in biological samples were flagged and removed. After these quality-control steps, the data matrix was normalised by Probabilistic Quotient Normalization (PQN) (Louail et al., 2025).

Metabolites were identified through a two-tiered approach following the Metabolomics Standards Initiative (MSI) guidelines. Level 1 identification (confident identification) was achieved by matching both retention time and MS/MS spectrum against an in-house library constructed from over 60 authentic standards representative of tea metabolites (including amino acids, catechins, flavonoid glycosides, phenolic acids, etc.), all analysed under identical LC-MS conditions. Matching criteria were: retention time tolerance ≤ 0.1 min, forward and reverse spectral match scores ≥ 800 (on a 0–999 scale), and precursor ion mass error ≤ 5 ppm. Level 2 identification (putative annotation) was applied to features lacking a matching standard: accurate mass-based annotation was performed against the HMDB and KEGG databases with a mass tolerance of <10 ppm, and the molecular formula was validated by isotopic distribution analysis. All identified metabolites, together with their observed *m/z*, mass error, retention time, match scores, MSI confidence level, and identification source, are listed in a supplementary table.

### 2.5 Sensory evaluation

The sensory evaluation was conducted by a professional panel consisting of 20 trained tea tasters (aged 23–61 years). The evaluation protocol strictly followed the Chinese National Standard GB/T 23776-2018 (Methodology for sensory evaluation of tea) (appearance 25%, liquor color 10%, aroma 25%, taste 30%, infused leaves 10%). Specifically, 3.0 g of each tea sample was infused in a covered tasting cup with 150 mL of boiling water for exactly 5 min. In order to further characterize the aroma and taste dimensions of GBT samples harvested in different seasons, an additional descriptive sensory analysis was conducted. Specifically, panelists evaluated the infusion and scored the intensity of taste and aroma on a 0–10 scale, where: 0-2 = very weak, 2-4 = weak, 4-6 = moderate, 6-8 = strong, and 8-10 = very strong (Zeng et al., 2023). Each sample was evaluated in triplicate, and data are expressed as mean values.

### 2.6 Statistical analyses

Raw data were preliminarily processed using Microsoft Excel 365. One-way analysis of variance (ANOVA) and multifactor ANOVA were performed using SPSS 24 software (SPSS Inc., Chicago, IL, USA) to evaluate significant differences among treatment groups (*P* < 0.05). Orthogonal partial least squares-discriminant analysis (OPLS-DA) was conducted using SIMCA 14.1 software (Umetrics, Umeå, Sweden), and variable importance in projection (VIP) values were calculated to identify key differential metabolites discriminating tea samples from different seasons (VIP > 1, *P* < 0.05). Metabolite correlation networks were constructed using Cytoscape 3.10.3 software (Cytoscape Consortium, Washington, DC, USA). Data visualization, including bar plots, stacked plots, radar charts, and heatmaps, was performed using the ChiPlot online tool (https://www.chiplot.online/).

## 3 Results and discussion

### 3.1 Sensory quality analysis

Sensory evaluation was conducted on GBT samples harvested in spring (SPT), summer (SMT), and autumn (AUT) (**Table 1**). The results indicated that the SPT exhibited the highest overall quality, with a significantly higher total score (94.77 ± 1.31) compared to the summer and autumn samples. SPT achieved the highest scores across all attributes: appearance (94.45 ± 3.31), liquor color (94.53 ± 2.87), aroma (95.33 ± 2.34), taste (94.95 ± 3.84), and infused leaves (93.93 ± 4.3). It was characterized by tightly twisted, heavy leaves with golden tips, an orange-red bright liquor, a rich sweet aroma with a slight cinnamon note, a fresh, strong, mellow and sweet taste, and fine, tender, reddish-bright infused leaves. The SMT received the lowest total score (90.34 ± 2.1), with significantly lower scores for liquor color (89.88 ± 5.56), aroma (88.98 ± 2.93), and taste (88.65 ± 5.63) compared to SPT, exhibiting a relatively light taste with astringency. The AUT obtained a moderate total score (92.15 ± 2.06). While their aroma score (93.65 ± 2.71) was comparable to SPT, featuring a sweet aroma with a high floral note and a slight cinnamon note, their appearance (92.2 ± 2.75) was somewhat coarse and their taste (92.13 ± 5.18) was slightly astringent.

**Table 1:** Sensory evaluation scores of spring, summer, and autumn GBT samples.

Samples	Appearance (25%)		Soup color (10%)		Aroma (25%)		Taste (30%)		Brewed leaves (10%)		Total score
	Score	Comment	Score	Comment	Score	Comment	Score	Comment	Score	Comment	
SPT	94.45 ± 3.31a	Dark brown with glossy sheen, tight and fine, uniform and clean, with golden tips, heavy and solid.	94.53 ± 2.87a	Orange-red and bright.	95.33 ± 2.34a	Rich sweet aroma, slight cinnamon note.	94.95 ± 3.84a	Fresh, strong, mellow and thick, sweet.	93.93 ± 4.3a	Fine and tender, reddish and bright.	94.77 ± 1.31a
SMT	93.2 ± 2.97ab	Fairly tightly twisted, black and glossy, well-proportioned, clean.	89.88 ± 5.56b	Red and bright.	88.98 ± 2.93b	Sweet aroma, average aroma.	88.65 ± 5.63b	Fresh, mellow, light, with astringency.	92.15 ± 2.89ab	Relatively tender, reddish and fairly bright.	90.34 ± 2.1c
AUT	92.2 ± 2.75b	Stout, fairly uniform, fairly clean, coarse.	89.88 ± 4.99b	Orange-yellow and fairly bright.	93.65 ± 2.71a	Sweet aroma, high floral note, slight cinnamon note.	92.13 ± 5.18a	Fresh and mellow, slightly astringent, fairly thick.	90.6 ± 4.25b	Fibrous, fairly reddish and bright.	92.15 ± 2.06b

Values are shown as mean ± SD (n=20). Different letters indicate statistically significant differences at *P* < 0.05.

**Figure 2:**
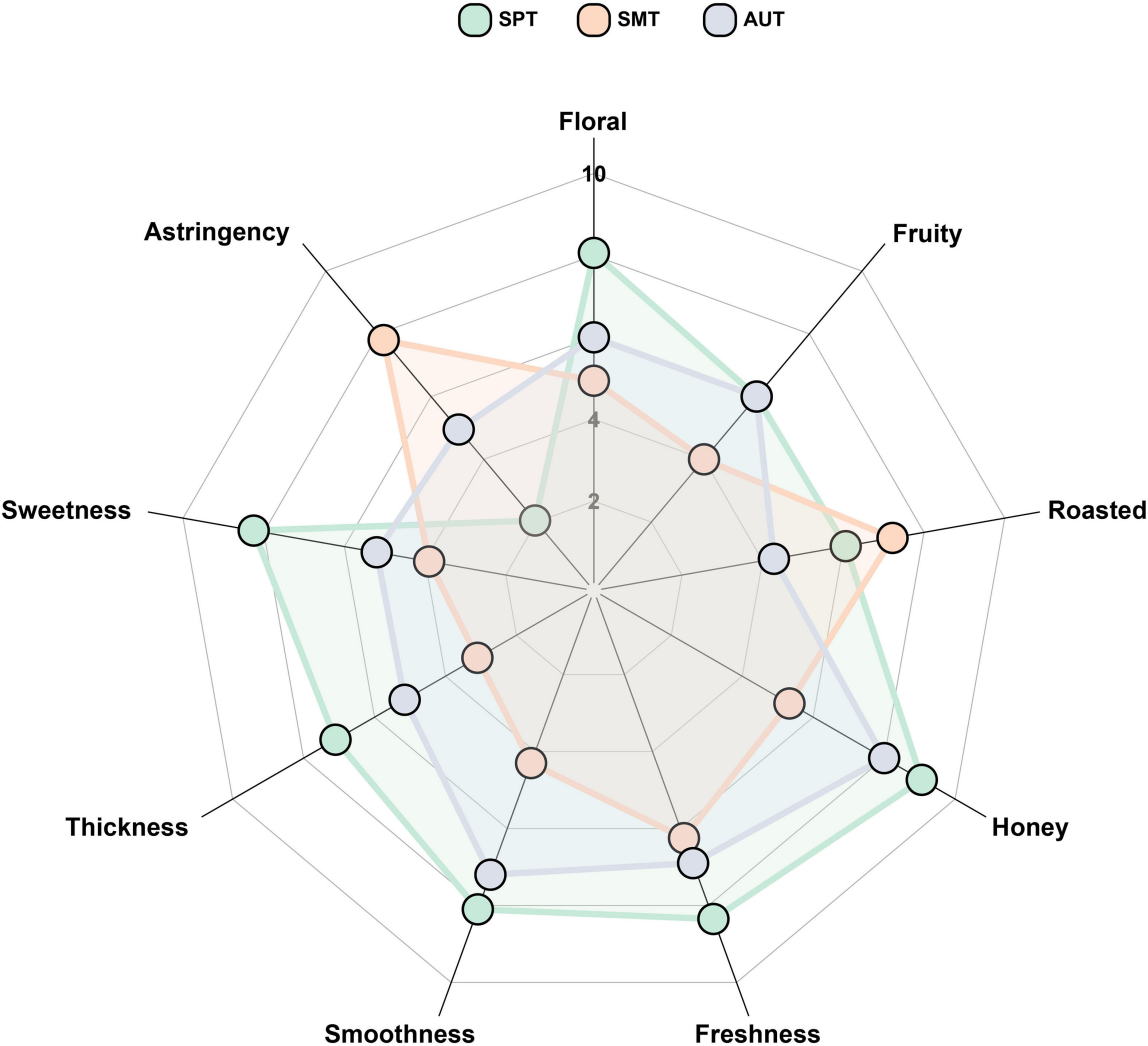
Sensory evaluation results of spring, summer, and autumn GBT, shown as a radar chart of aroma and taste attributes.

A 0-to-10 scale was employed to evaluate aroma and taste attributes, where scores of 0–2, 2–4, 4–6, 6–8, and 8–10 represented very weak, weak, moderate, strong, and extremely strong intensity, respectively. The results are presented in **Supplementary Table 1** and visualized as a radar chart in **Figure 2**. Regarding aroma, SPT exhibited extremely high intensities of floral (8.06) and honey-like (9.06) notes, with fruity and roasty attributes at moderately high levels (both scored 6). SMT was characterized by a relatively pronounced roasty note (7.22), but low floral (4.94) and fruity (4) intensities, along with a moderate-to-low honey-like note (5.33). AUT showed a high honey-like intensity (8), moderate floral and fruity notes (both scored 6), and a weak roasty intensity (4.28). In terms of taste, SPT displayed high levels of freshness, smoothness, and sweetness (scores of 8.00, 8.00, and 8.00, respectively), a relatively high thickness (7.80), and extremely low astringency (2.05). SMT exhibited strong astringency (7.80), moderate freshness (6.25), but low smoothness (4.30), thickness (3.10), and sweetness (3.90). AUT presented a more balanced taste profile, with moderately high freshness (6.90) and smoothness (7.20), and moderate levels of thickness (5.15), sweetness (5.20), and astringency (4.95).

### 3.2 Volatile metabolome analysis

#### 3.2.1 Identification and clustering of volatile metabolites

**Figure 3:**
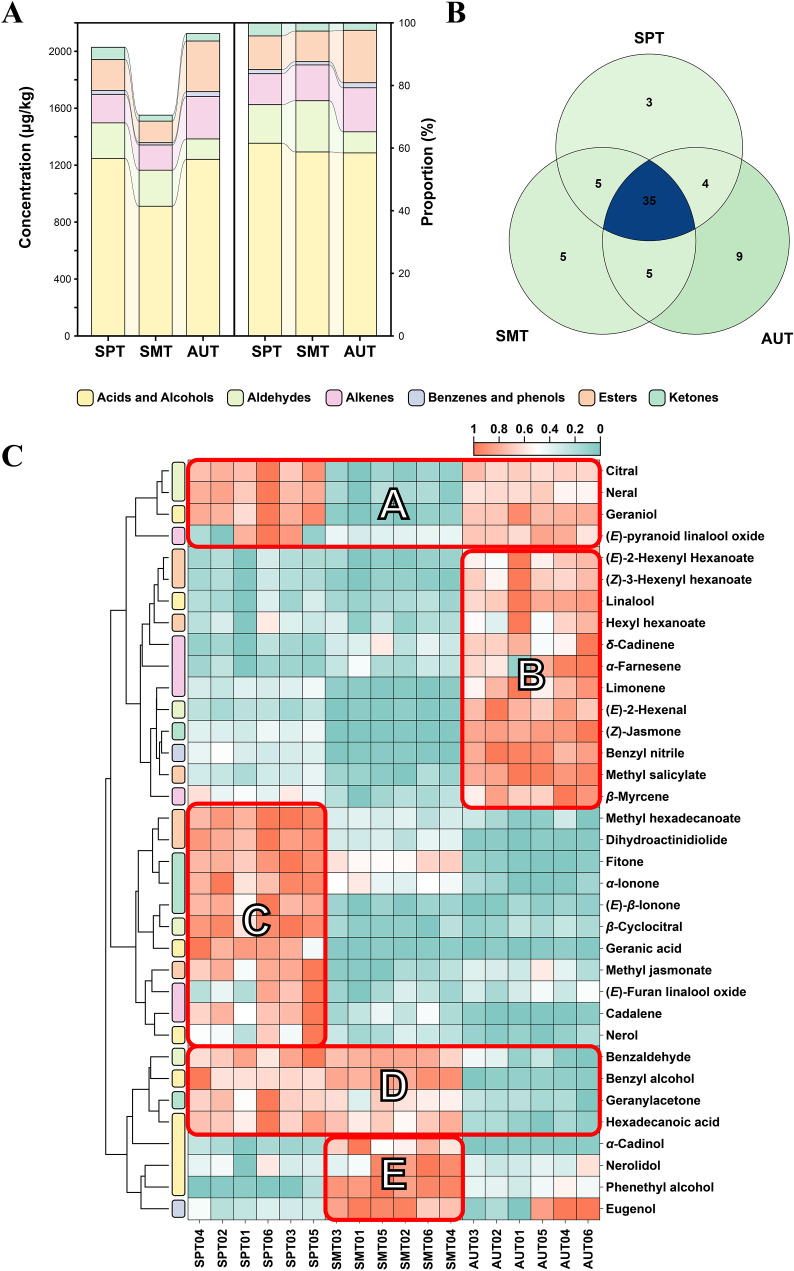
Qualitative and quantitative analysis of volatile compounds in GBT harvested in different seasons. **(A)** Stacked bar chart showing the contents of various volatile classes. **(B)** Venn diagram of volatile compounds. **(C)** Heatmap of common volatile compounds.

A total of 66 volatile metabolites were identified, with 47, 53, and 50 compounds detected in spring, summer, and autumn teas, respectively (**Supplementary Table 2**). Chromatograms graphs can be seen in **Supplementary Figure 1**. The total volatile content was highest in AUT (2122.79μg/kg), followed by SPT (2026.40μg/kg) and SMT (1549.52μg/kg) teas (**Figure 3A, B**). Based on functional group classification, these compounds comprised acids and alcohols (12), aldehydes (11), alkenes (14), benzenes and phenols (5), esters (18), and ketones (5). A total of 35 volatile compounds were commonly detected across all three seasons, and their relative abundance profiles were visualized by heatmap analysis as shown in **Figure 3C**.

Based on heatmap clustering analysis, 66 volatile metabolites were categorized into five distinct accumulation patterns (Clusters A–E) according to their content variations across spring, summer, and autumn teas. The seasonal distribution characteristics of each cluster are as follows:

Cluster A comprised four monoterpenoid derivatives—citral, (*E*)-pyranoid linalool oxide, geraniol, and neral—which exhibited higher accumulation in spring and autumn but lower levels in summer. Cluster B contained twelve compounds, including *β*-myrcene, methyl salicylate, benzyl nitrile, (*Z*)-jasmone, (*E*)-2-hexenal, limonene, *α*-farnesene, *δ*-cadinene, hexyl hexanoate, linalool, (*Z*)-3-hexenyl hexanoate, and (*E*)-2-hexenyl hexanoate, all of which peaked in AUT. Cluster C consisted of eleven metabolites—nerol, cadalene, (*E*)-furan linalool oxide, methyl jasmonate, geranic acid, *β*-cyclocitral, (*E*)-*β*-ionone, *α*-ionone, fitone, dihydroactinidiolide, and methyl hexadecanoate—with the highest levels observed in SPT. Cluster D included hexadecanoic acid, geranylacetone, benzyl alcohol, and benzaldehyde, showing elevated accumulation in both spring and summer but lower levels in autumn. Cluster E contained eugenol, phenethyl alcohol, nerolidol, and *α*-cadinol, which were specifically accumulated in SMT.

The differential accumulation patterns of volatile metabolites observed above provide metabolic insights into the seasonal variations in sensory quality of GBTs. Specifically, the predominance of monoterpenoids (Cluster A) and norisoprenoids (Cluster C) in SPT—such as geraniol, *β*-ionone, and dihydroactinidiolide—can be attributed to their low odor thresholds and high aromatic impact (Kumazawa and Masuda, 2002; Hattori et al., 2003; Xu et al., 2018), collectively underpinning the “rich sweet aroma” and “fresh, mellow taste” characteristic of spring-harvested samples (Yu et al., 2023). In contrast, the specific accumulation of Cluster B compounds in AUT, predominantly fatty acid-derived green leaf volatiles and inducible defense terpenes, points to ecological adaptation. Notably, *α*-farnesene and benzyl nitrile have been implicated in herbivore-induced defense responses in tea plants, suggesting that AUT shoots may encounter specific biotic pressures, thereby drive the accumulation of these volatiles and contributing to the complex “floral and fruity” notes of AUT (Zeng et al., 2017; Qian et al., 2023). Conversely, the exclusive accumulation of Cluster E compounds (eugenol, nerolidol, etc.) in SMT aligns with its pronounced astringency (score 8) in sensory evaluation. Heat and light stress during summer months have been demonstrated to activate the phenylpropanoid pathway, leading to increased phenolic accumulation and enhanced astringency (Zou et al., 2022); concurrently, stress-induced upregulation of sesquiterpene biosynthesis may further modulate aroma harmony (Gao et al., 2025). Thus, the flavor profile of SMT can be interpreted as a metabolic consequence of the tea plant’s adaptive response to environmental stress.

To further visualize the separation of volatile profiles among the three seasons, PCA was performed on the identified volatile compounds (**Supplementary Figure 2**). The results showed a clear clustering of SPT, SMT, and AUT samples, indicating distinct seasonal volatile patterns.

#### 3.2.2 Screening of season-differential volatile compounds

To identify differential volatile compounds discriminating GBTs harvested in spring, summer, and autumn, an orthogonal partial least squares discriminant analysis (OPLS-DA) model was established. The model parameters were R²X=0.898 and R²Y=0.98, and validation via 200 permutation tests confirmed no overfitting (Q²=0.427), indicating good stability and predictive ability for differential volatile screening (**Supplementary Figure 3**).

**Figure 4:**
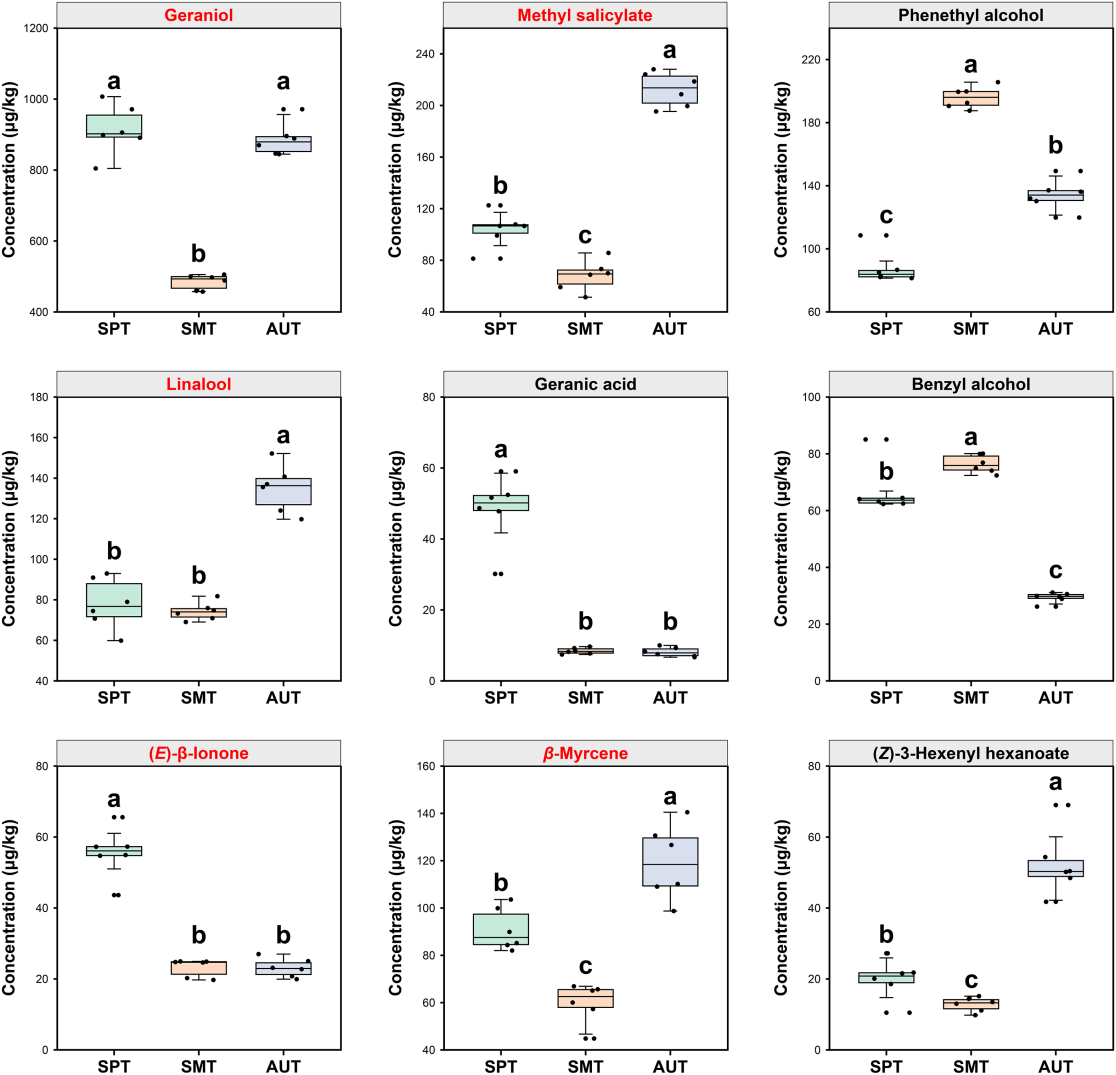
Key volatile compounds of 3 GBTs in different seasons. Different letters indicate significant differences at *P* < 0.05.

Using VIP>1 combined with *P* < 0.05 as the threshold, nine volatile compounds with significant seasonal differences were identified (**Supplementary Table 3**). Further evaluation based on odor activity values (rOAV>1) revealed that five compounds—geraniol, methyl salicylate, linalool, (*E*)-*β*-ionone, and *β*-myrcene—satisfied both VIP>1 and rOAV>1 criteria, confirming their roles as key contributors to the seasonal aroma characteristics of GBTs (**Figure 4**).

Regarding seasonal accumulation patterns, these nine differential compounds exhibited distinct profiles: (1) Geranic acid and (*E*)-*β*-ionone were specifically accumulated in SPT; (2) Methyl salicylate, linalool, *β*-myrcene, and (*Z*)-3-hexenyl hexanoate were specifically accumulated in AUT; (3) Geraniol showed high accumulation in both SPT and AUT; (4) Phenethyl alcohol and benzyl alcohol were specifically accumulated in SMT.

Among the nine VIP>1 differential compounds, five simultaneously satisfied rOAV>1, indicating that these compounds serve not only as chemical markers for seasonal discrimination but also as key contributors to the perceived aroma of GBTs. Notably, the seasonal distribution of these high-rOAV compounds aligned closely with sensory evaluation results: (*E*)-*β*-ionone, accumulated specifically in SPT, possesses an extremely low odor threshold (0.007 μg/L) and a characteristic violet-like aroma; its synergistic interaction with geraniol has been demonstrated to enhance sweet aroma perception in tea (Deng et al., 2024; Wei et al., 2026). Linalool, *β*-myrcene, and methyl salicylate, specifically accumulated in AUT, are key products of fatty acid/terpene metabolic pathways (Qiao et al., 2022). Among these, methyl salicylate functions as a signaling molecule in plant defense responses, and its high accumulation in autumn may be associated with environmental stress induction.

Geraniol, the only compound highly accumulated in both spring and autumn, is regulated by alternative splicing of the *CsbHLH133* gene in tea plants—under stress conditions, the *CsbHLH133-AS* splice variant becomes the predominant transcript, modulating geraniol biosynthesis to adapt to unfavorable environments (Cheng et al., 2024). This mechanism explains why geraniol content decreases under summer stress conditions while accumulating efficiently during the favorable climatic conditions of spring and autumn.

Phenethyl alcohol and benzyl alcohol, specifically accumulated in SMT, are products of the phenylpropanoid pathway. Their accumulation pattern correlates with the pronounced astringency (score 8) observed in sensory evaluation of SMT. Research indicates that high temperature and strong light stress during summer activate the phenylpropanoid pathway, leading to phenolic compound accumulation, while simultaneously inhibiting terpene biosynthesis (Li et al., 2022; Gao et al., 2025). This stress-induced metabolic reprogramming explains the phenomenon where SMT exhibits a limited aroma profile (only phenylpropanoids highly accumulated) with generally low terpene levels.

OPLS-DA modeling combined with VIP and rOAV analysis successfully identified nine seasonal differential volatile compounds, with five confirmed as key contributors to the seasonal aroma characteristics of GBTs. The season-specific accumulation patterns of these compounds reflect differential regulation of terpene synthesis, fatty acid metabolism, and phenylpropanoid pathways in tea plants under varying environmental conditions, thereby elucidating the formation mechanisms underlying the distinct sensory qualities of spring, summer, and autumn GBTs at the molecular level.

### 3.3 Correlation analysis of volatile compounds with aroma attributes

**Figure 5:**
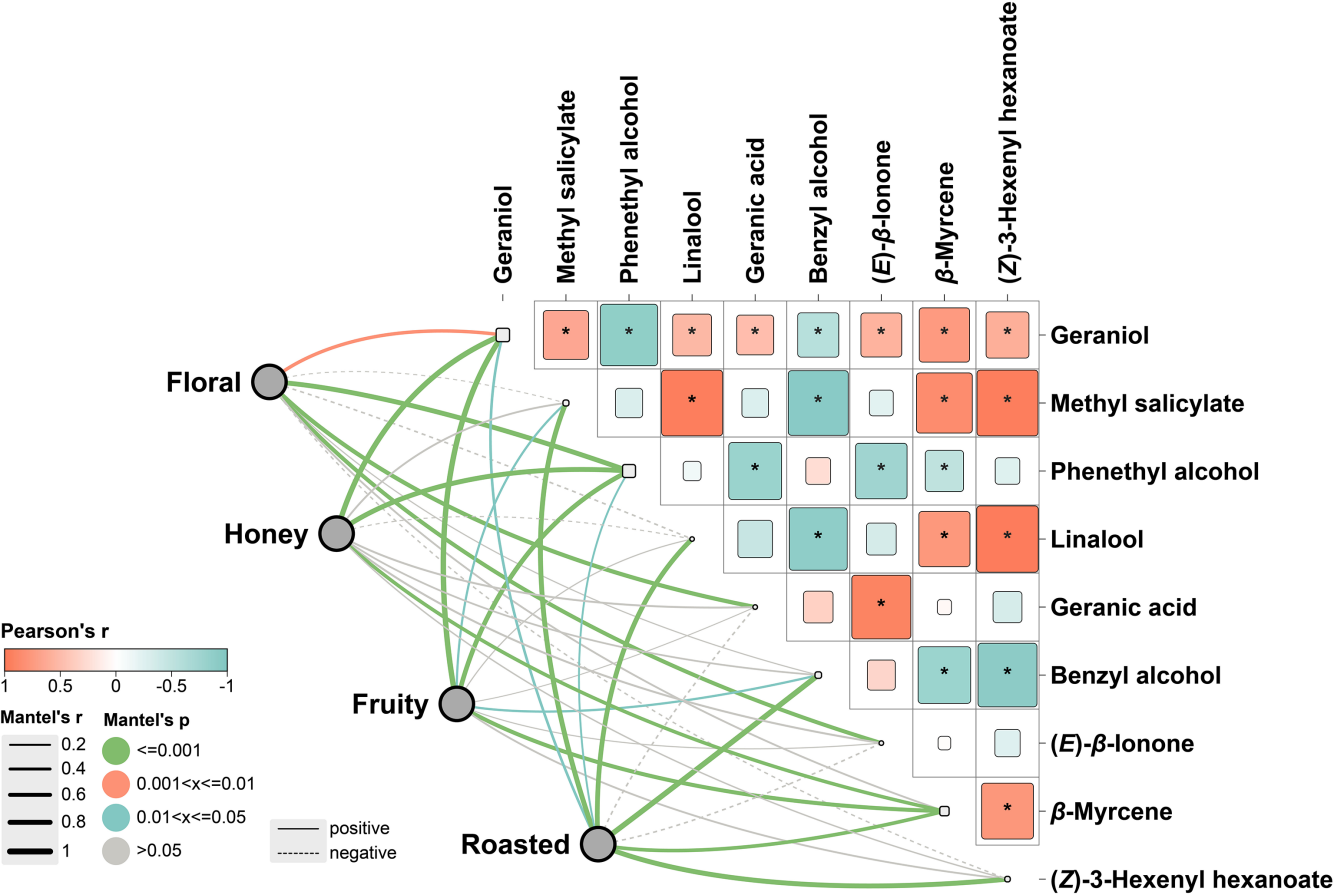
Correlation network diagram between key aroma-active compounds and sensory aroma attributes. Statistical significances are denoted by * for p < 0.05.

To explore the potential factors underlying seasonal variations in tea quality, Pearson correlation analysis was conducted between nine key differential volatile compounds and four sensory attributes (**Figure 5**). The results revealed several significant positive correlations (*P* < 0.05) between specific volatiles and sensory descriptors. Geraniol, phenethyl alcohol, (*E*)-*β*-ionone, and geranic acid showed significant positive correlations with the floral attribute, suggesting these compounds as potential key contributors to the floral aroma characteristic. Additionally, geraniol, phenethyl alcohol, and *β*-myrcene exhibited significant positive correlations with the honey-like attribute, indicating their synergistic roles in shaping the overall sweet, fruity, and floral dimensions of GBT. It should be noted that these correlations represent associative trends rather than definitive causal relationships, as aroma perception inherently arises from complex interactions among multiple volatile components.

The correlation patterns observed in this study are aligned with recent findings in tea flavor chemistry. Geraniol and (*E*)-*β*-ionone have been identified as common odor-active compounds across multiple black tea varieties, contributing significantly to floral and sweet aroma perceptions (Wei et al., 2025; Liu et al., 2022). Notably, Deng et al. demonstrated that (*E*)-*β*-ionone and geraniol exhibit additive synergistic interactions in binary mixture models, significantly enhancing sweet aroma intensity through cross-modal perception mechanisms mediated by the central nervous system (Deng et al., 2024). Furthermore, phenethyl alcohol has been positively correlated with floral and sweet honey aroma intensities in Hunan black tea, further supporting its potential contribution to the honey-like dimension in GBT (Yin et al., 2022). These findings suggest that the characteristic aroma formation in GBT results from synergistic interactions among multiple key odorants rather than the action of individual compounds.

### 3.4 Non-volatile metabolome analysis

#### 3.4.1 Multivariate statistical analysis of non-volatile metabolites

An untargeted metabolomics approach was employed to comprehensively investigate the non-volatile metabolite profiles of GBTs harvested in different seasons. Based on UPLC-MS/MS analysis and an in-house database, a total of 9894 ion features were identified, encompassing major categories including lipids and lipid-like molecules, phenylpropanoids and polyketides, organoheterocyclic compounds, organic acids and derivatives, benzenoids, organic oxygen compounds, nucleosides and analogues, and alkaloids and derivatives.

PCA was first applied to evaluate the overall clustering of non−volatile metabolite profiles across different seasons (**Supplementary Figure 4**). The score plot revealed a distinct separation among SPT, SMT, and AUT samples, supporting the presence of substantial seasonal metabolic differences. Subsequently, OPLS-DA was performed on the identified metabolites. The model exhibited excellent explanatory and predictive ability (R²Y=0.993, Q²=0.987), and validation via 200 permutation tests confirmed its robustness, as the regression line of permuted Q² values intersected the vertical axis below zero, indicating no overfitting (**Supplementary Figure 5A, B**).

Using VIP>1 and *P ≤* 0.05 as thresholds, 950 key differential metabolites were identified (**Supplementary Table 4**), and a heatmap analysis of them can be seen in **Supplementary Figure 6**. Based on chemical classification, these comprised primarily lipids and lipid-like molecules (313), phenylpropanoids and polyketides (194), organoheterocyclic compounds (166), organic acids and derivatives (106), benzenoids (74), and organic oxygen compounds (44). To further prioritize the most biologically relevant compounds from the 950 identified differential metabolites, we performed a ranking-based selection within each major chemical subclass. Specifically, for each subclass, the top 20 metabolites with the highest VIP values (VIP > 1) and the most significant differential expression (P ≤ 0.05) were selected, including flavonoid glycosides (23), amino acids and derivatives (18), benzoic acids and derivatives (13), biflavonoids and polyflavonoids (10), flavans (10), and triterpenoids (8) (**Supplementary Figure 5C**).

**Figure 6:**
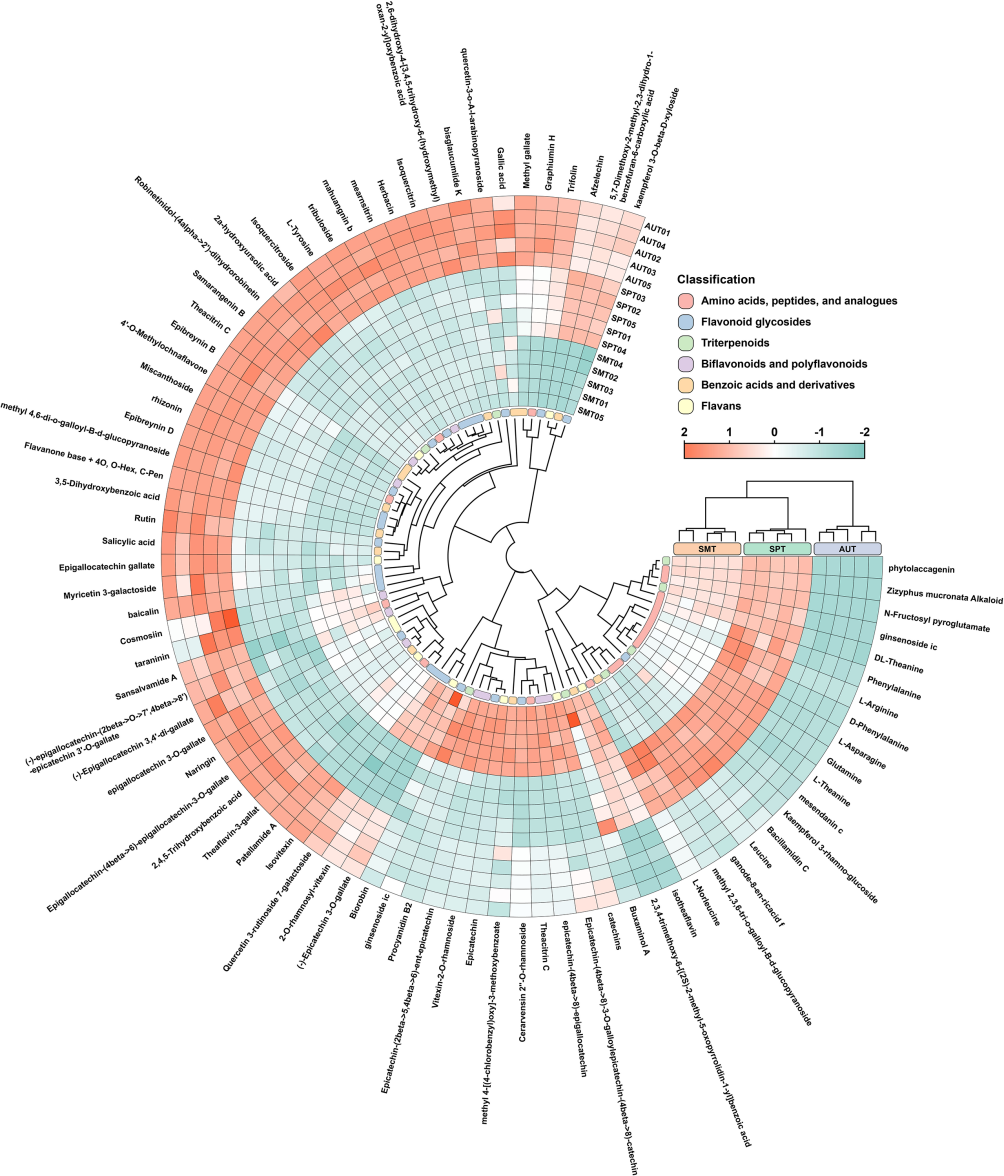
Heatmap clustering analysis of 82 key differential non-volatile metabolites in GBTs harvested in different seasons.

Heatmap clustering analysis of the 82 key differential metabolites revealed distinct metabolic profiles among seasonal samples (**Figure 6**) (**Supplementary Table 5**). In SPT, amino acids and their analogues, including theanine (DL-Theanine and L-Theanine)—key contributors to umami taste—were significantly up-regulated, aligning with the pronounced freshness perceived in sensory evaluation. In SMT, most metabolites exhibited the lowest abundances across the three seasons, with only a subset of flavonoid glycosides showing up-regulation, consistent with its relatively bitter and astringent taste quality. In AUT, most compounds, including flavonoid glycosides, triterpenoids, biflavonoids and polyflavonoids, benzoic acids and derivatives, and flavans, displayed up-regulated trends, providing the metabolic basis for its rich and mellow taste profile.

The seasonal accumulation patterns of these metabolites elucidate the biochemical basis underlining the quality formation of GBTs. The specific accumulation of theanine in SPT can be attributed to the seasonal regulation of its biosynthesis—*CsGDH2.1* negatively regulates theanine accumulation via modulating glutamate metabolism, with its low expression level in early spring facilitating theanine enrichment (Chen et al., 2023). The accumulation pattern of flavonol glycosides in SMT aligns with findings on Lu’an Guapian tea (Li et al., 2025), indicating that flavonoid biosynthesis is a key pathway responsible for seasonal quality variations. Zhang et al (Zhang et al., 2023). also confirmed in summer Keemun black tea that variations in flavonoid content directly correlate with changes in bitterness and astringency. The synergistic accumulation of multiple flavonoids and triterpenoids in AUT explains its characteristic mellow and full-bodied taste, corroborating reports by Zhou et al (Zhou et al., 2025). on the impact of seasonality on oolong tea quality.

#### 3.4.2 Metabolic pathway analysis of differential metabolites

**Figure 7:**
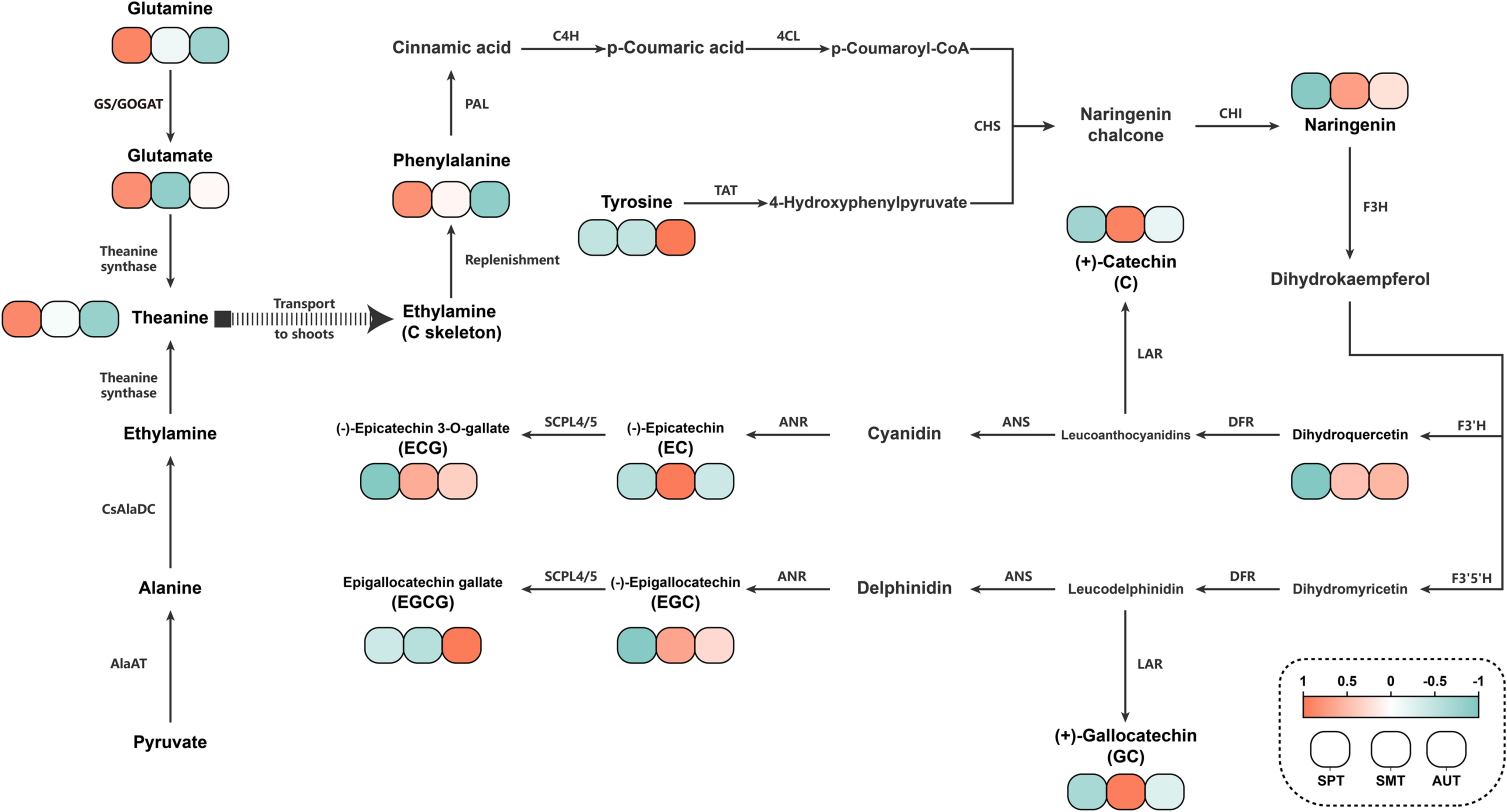
Simplified schematic diagram of the biosynthetic pathways of theanine, phenylalanine, and catechins (esterified and non-esterified).

To elucidate the metabolic basis underlying seasonal quality variations in GBT, pathway enrichment analysis was performed on 82 key differential metabolites (**Supplementary Figure 7**). The results revealed significant enrichment in phenylalanine, tyrosine and tryptophan biosynthesis, phenylalanine metabolism, arginine biosynthesis, alanine, aspartate and glutamate metabolism, valine, leucine and isoleucine biosynthesis, and D-amino acid metabolism (*P* < 0.05). Based on the simplified schematic model of theanine and catechin biosynthesis pathways (**Figure 7**), the detected metabolites exhibited season-specific accumulation patterns: SPT exhibited the highest abundances of glutamine, glutamate, theanine, and phenylalanine, underscoring its central role in nitrogen metabolism. SMT was characterized by the pronounced accumulation of flavan-3-ols, including catechin (C), gallocatechin (GC), epicatechin (EC), epicatechin gallate (ECG), and epigallocatechin (EGC). AUT showed the highest levels of tyrosine and dihydroquercetin, indicating an enhanced metabolic flux through specific phenylpropanoid branches during seasonal transition.

The seasonal accumulation patterns of these metabolites reveal differential regulation of primary and secondary metabolic pathways in response to environmental factors (**Figure 7**). The high accumulation of theanine and related amino acid precursors in SPT can be attributed to seasonally enhanced nitrogen metabolism—the “root-to-shoot” transport of theanine is mediated by CsAAPs transporters (Luo and He, 2025), with favorable spring temperatures and light conditions facilitating theanine enrichment in newly formed shoots. The specific accumulation of ester catechins in SMT is closely associated with heat stress-induced upregulation of phenylpropanoid metabolism, consistent with previous reports of increased phenolic compound concentrations in summer-harvested teas (Fu et al., 2024). The accumulation of tyrosine and dihydroquercetin in AUT reflects metabolic flux redirection within the phenylalanine pathway during seasonal transition. These findings elucidate the molecular basis underlying the distinct quality characteristics of spring, summer, and autumn GBTs from a metabolic flux perspective.

#### 3.4.3 Correlation analysis of differential metabolites with taste attributes

**Figure 8:**
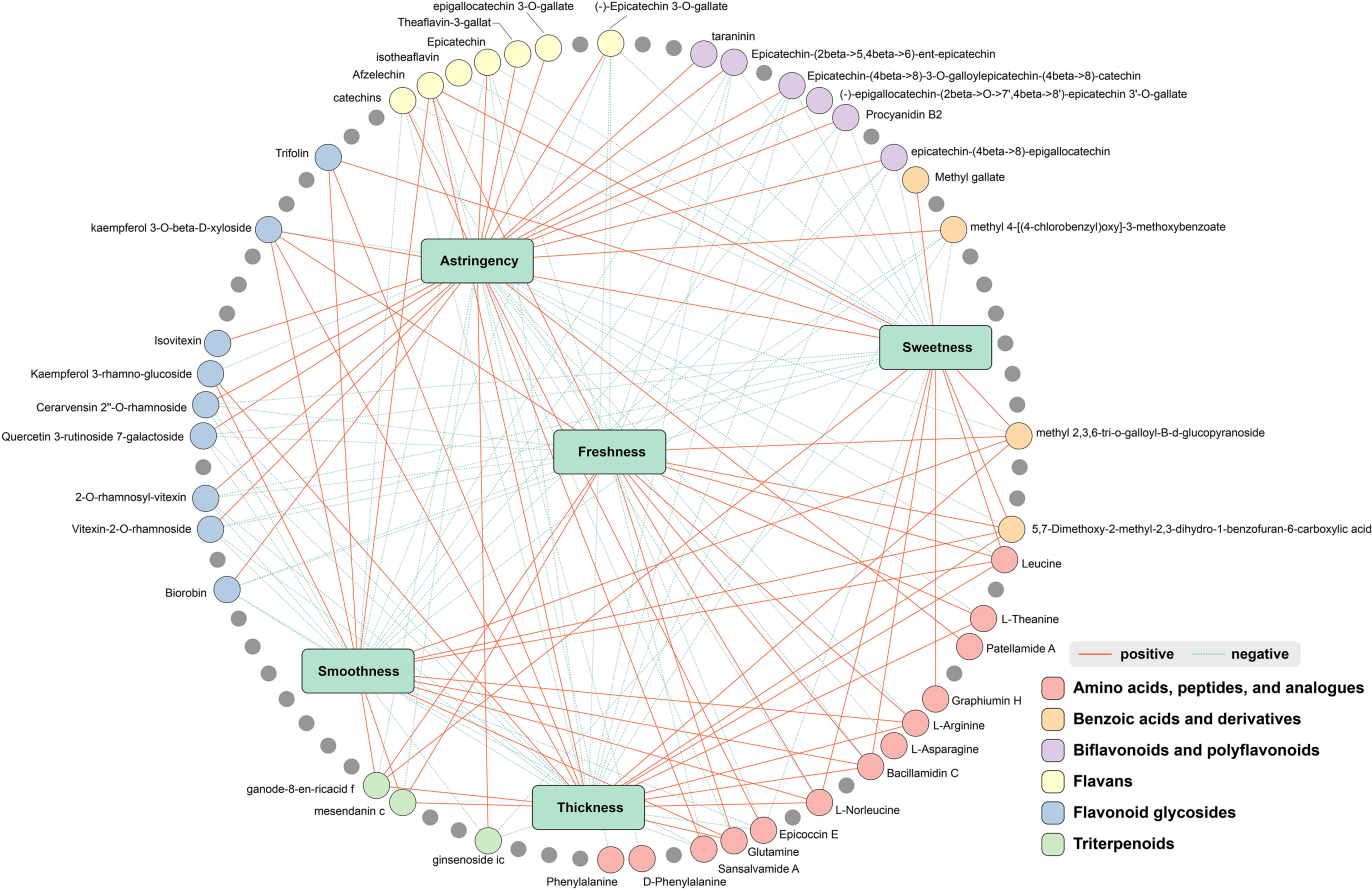
Association networks linking key non-volatile metabolites with sensory taste attributes. Square nodes represent the five sensory taste attributes, and circular nodes represent individual metabolites. Gray nodes indicate no significant correlation with the taste attributes, while colored nodes denote significant correlations, with colors corresponding to different metabolite classes. Solid and dashed lines represent positive and negative correlations, respectively.

To elucidate the associations between key differential metabolites and taste attributes, Pearson correlation analysis was performed between 82 metabolites and five sensory descriptors—freshness, smoothness, thickness, sweetness, and astringency—followed by construction of a correlation network (**Figure 8**) (**Supplementary Tables 6**, **S7**). The results revealed a pronounced class-dependent pattern in the metabolite-taste association network.

Amino acids and their derivatives generally exhibited significant positive correlations with favorable taste attributes. L-Theanine showed significant positive correlations with freshness and sweetness, along with a negative correlation with astringency. Leucine demonstrated significant positive correlations with all five favorable attributes. L-Arginine was positively correlated with freshness, thickness, and sweetness. L-Asparagine showed positive correlation with sweetness. These findings align with recent studies—Narukawa et al (Narukawa et al., 2014). confirmed that L-theanine elicits umami perception through activation of the T1R1/T1R3 umami receptor. Deng et al.’s research on Longjing tea further revealed that L-theanine and L-arginine synergistically enhance umami intensity (Deng et al., 2025). Regarding the regulatory mechanism of theanine biosynthesis, recent discoveries demonstrate that gibberellin promotes theanine accumulation by suppressing *CsWRKY71* expression, a transcription factor that inhibits the key biosynthetic gene *CsTSI* (Xiang et al., 2025).

Flavonoid compounds exhibited contrasting correlation patterns. Quercetin 3-rutinoside 7-galactoside, 2-O-rhamnosyl-vitexin, Vitexin-2-O-rhamnoside, Biorobin, and various polyflavanoids all showed significant positive correlations with astringency, while displaying significant negative correlations with freshness, smoothness, thickness, and sweetness. Notably, kaempferol 3-O-beta-D-xyloside showed significant positive correlations with all five favorable attributes, and Afzelechin displayed a similar pattern, suggesting that glycosylation patterns or polymerization degrees may modulate taste perception direction. Roland et al.’s study (Roland et al., 2013)demonstrated that flavonoids primarily elicit bitter-astringent perception through activation of bitter taste receptors hTAS2R14 and hTAS2R39. Zhang et al.’s review (Zhang et al., 2020) highlighted that flavonoid glycosides not only impart astringency to tea infusion but also enhance the bitterness perception of caffeine. Recent research from the Tea Research Institute of the Chinese Academy of Agricultural Sciences revealed that during spring warming, temperature influences the accumulation of bitter compounds such as quercetin glycosides by regulating gene expression including F3’5’H, thereby affecting tea bitterness quality (Yu et al., 2025).

Triterpenoids and organic acid derivatives displayed diverse association patterns. Ganode-8-en-ricacid f and mesendanin c showed positive correlations with freshness, thickness, and sweetness. Methyl 2, 3, 6-tri-o-galloyl-*β*-d-glucopyranoside exhibited significant positive correlations with all five favorable attributes, suggesting these compounds may contribute to tea’s mouthfeel and huigan (sweet aftertaste). Wang et al. found that Maillard reaction products of theanine and D-galacturonic acid produce taste effects through binding with umami receptors and the sour taste protein OTOP1 (Wang et al., 2024).

The balance between amino acids and flavonoids, together with their structural diversity, shapes the distinct taste quality characteristics of spring, summer, and autumn GBTs. Amino acids dominate favorable taste attributes including freshness, sweetness, and thickness through umami receptor activation, while flavonoid compounds primarily contribute to astringency through bitter taste receptor activation and salivary protein interactions. The seasonal differential accumulation of these two compound classes elucidates the molecular basis underlying the taste quality variations of teas harvested in different seasons.

## 4 Conclusions

This study systematically characterizes the flavor profiles underlying the sensory quality variations of spring, summer, and autumn GBTs by integrating sensory evaluation, volatile metabolomics, and untargeted metabolomics approaches. Sensory evaluation revealed that spring tea exhibited the optimal overall quality, characterized by rich sweet aroma and fresh, mellow taste; autumn tea presented complex aroma with harmonious floral and fruity notes; summer tea was distinguished by pronounced astringency and limited aroma complexity. A total of 66 volatile metabolites were identified, with nine compounds showing significant seasonal differences. Among these, five compounds—geraniol, methyl salicylate, linalool, (*E*)-*β*-ionone, and *β*-myrcene—simultaneously satisfied VIP>1 and rOAV>1 criteria, confirming their roles as key contributors to the seasonal aroma characteristics of GBT. Non-volatile metabolomics analysis identified 9894 ion features, with 82 core differential compounds screened. Amino acids accumulated predominantly in spring tea, dominating favorable taste attributes including freshness and sweetness. Flavonoids exhibited differential accumulation patterns in summer and autumn teas, closely associated with astringency formation. Pathway enrichment analysis identified phenylpropanoid metabolism, amino acid metabolism, and flavonoid biosynthesis as key pathways regulating seasonal quality variations. Correlation analysis further revealed a metabolite-taste association network, where amino acids positively correlated with favorable taste attributes while flavonoids positively correlated with astringency. In conclusion, this study elucidated at the molecular level how environmental factors differentially regulate primary and secondary metabolic pathways to shape the characteristic quality profiles of spring, summer, and autumn GBTs, providing initial insights for seasonal quality regulation and targeted production techniques of tea.

## Data Availability

The original contributions presented in the study are included in the article/**Supplementary Material**. Further inquiries can be directed to the corresponding author.
