# Supplementary figures and images for: Metabolomics-based elucidation of the chemical basis under-lying seasonal variations in sensory quality of Goldsands Black Tea
Source: Front Plant Sci. 2026 Jul 1;17:1888622. doi: 10.3389/fpls.2026.1888622 (PMC13369139; doi:10.3389/fpls.2026.1888622)

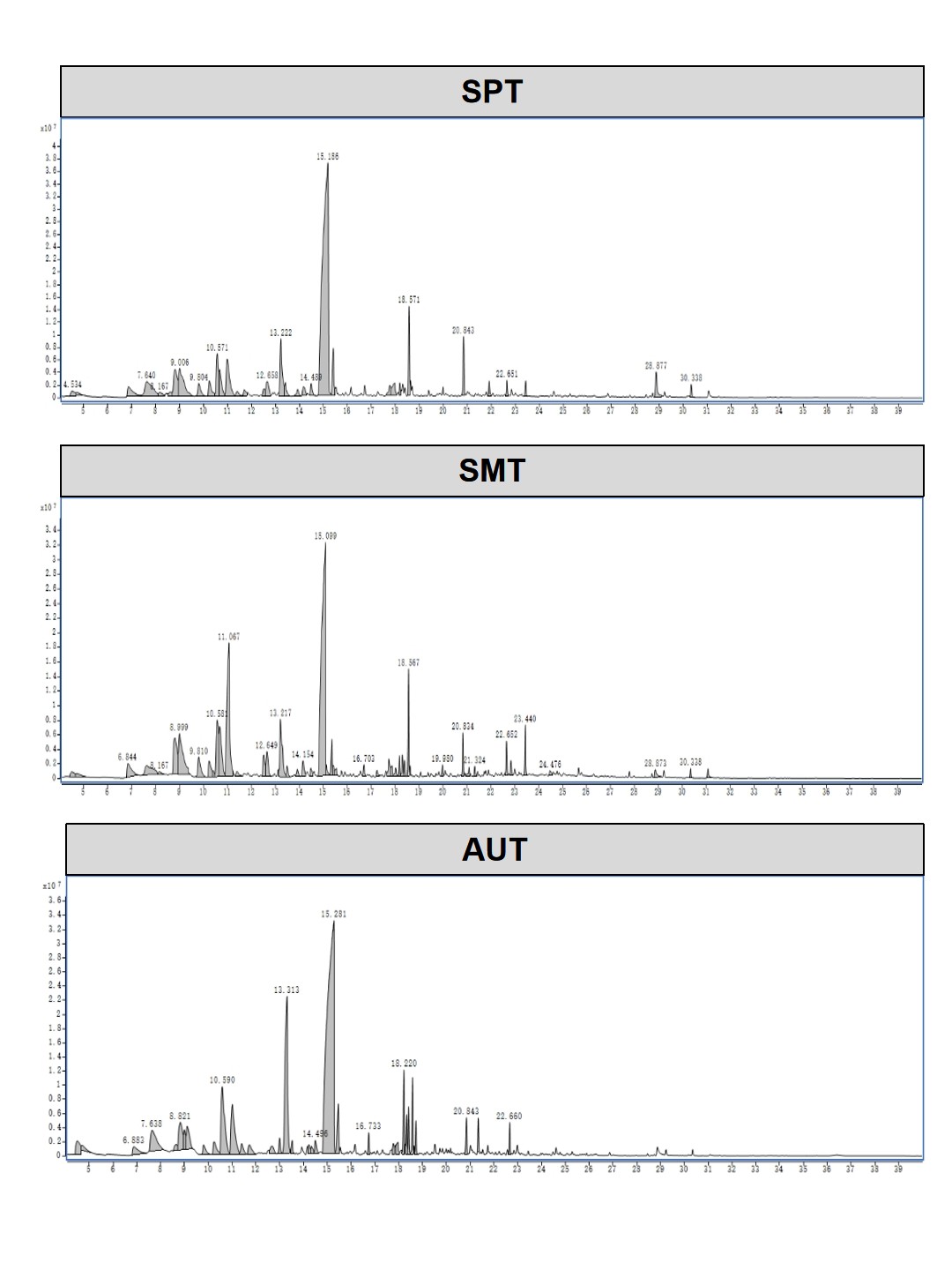

Supplement: Supplementary file 1 [file Image1.jpeg]

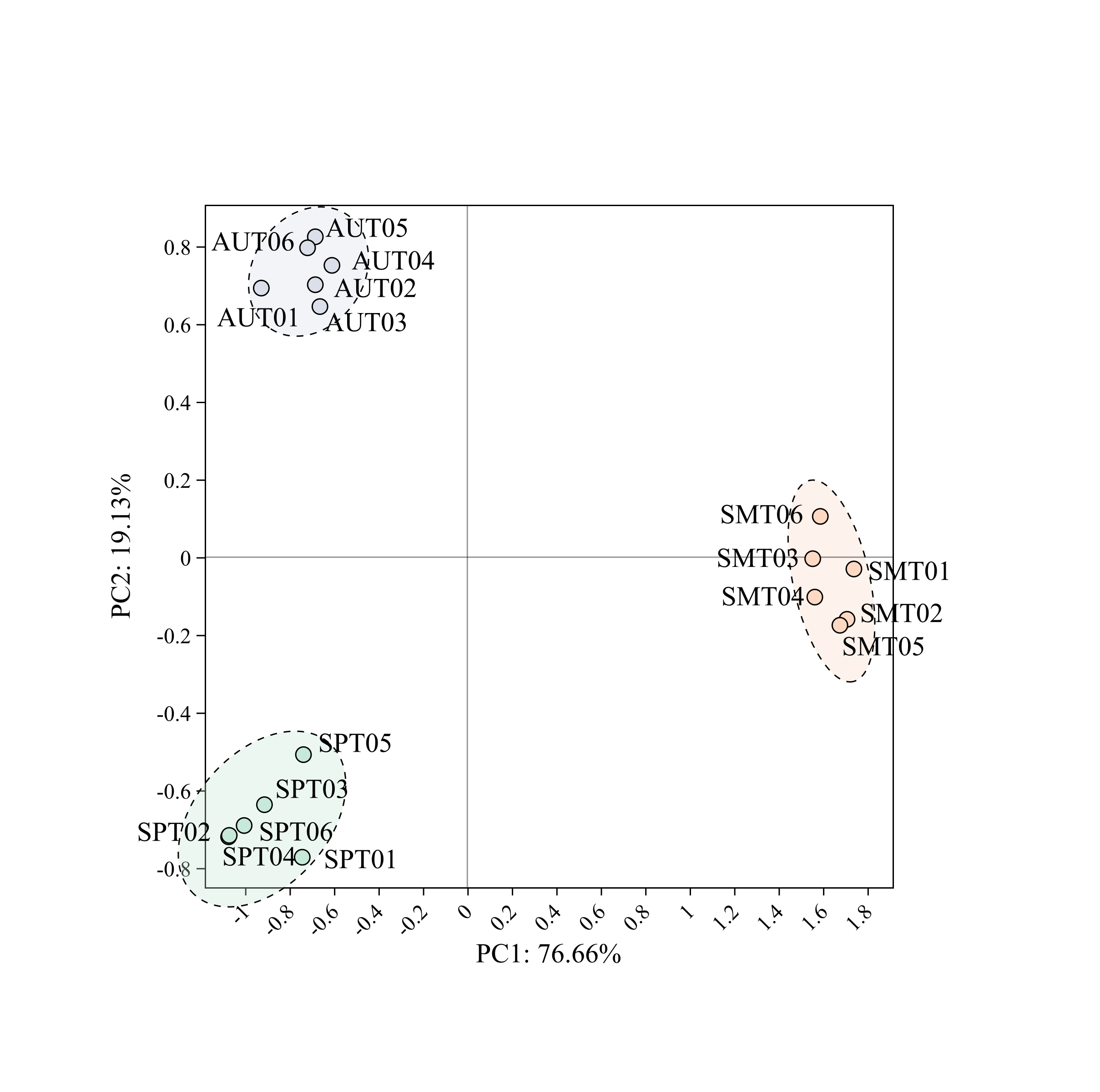

Supplement: Supplementary file 2 [file Image2.png]

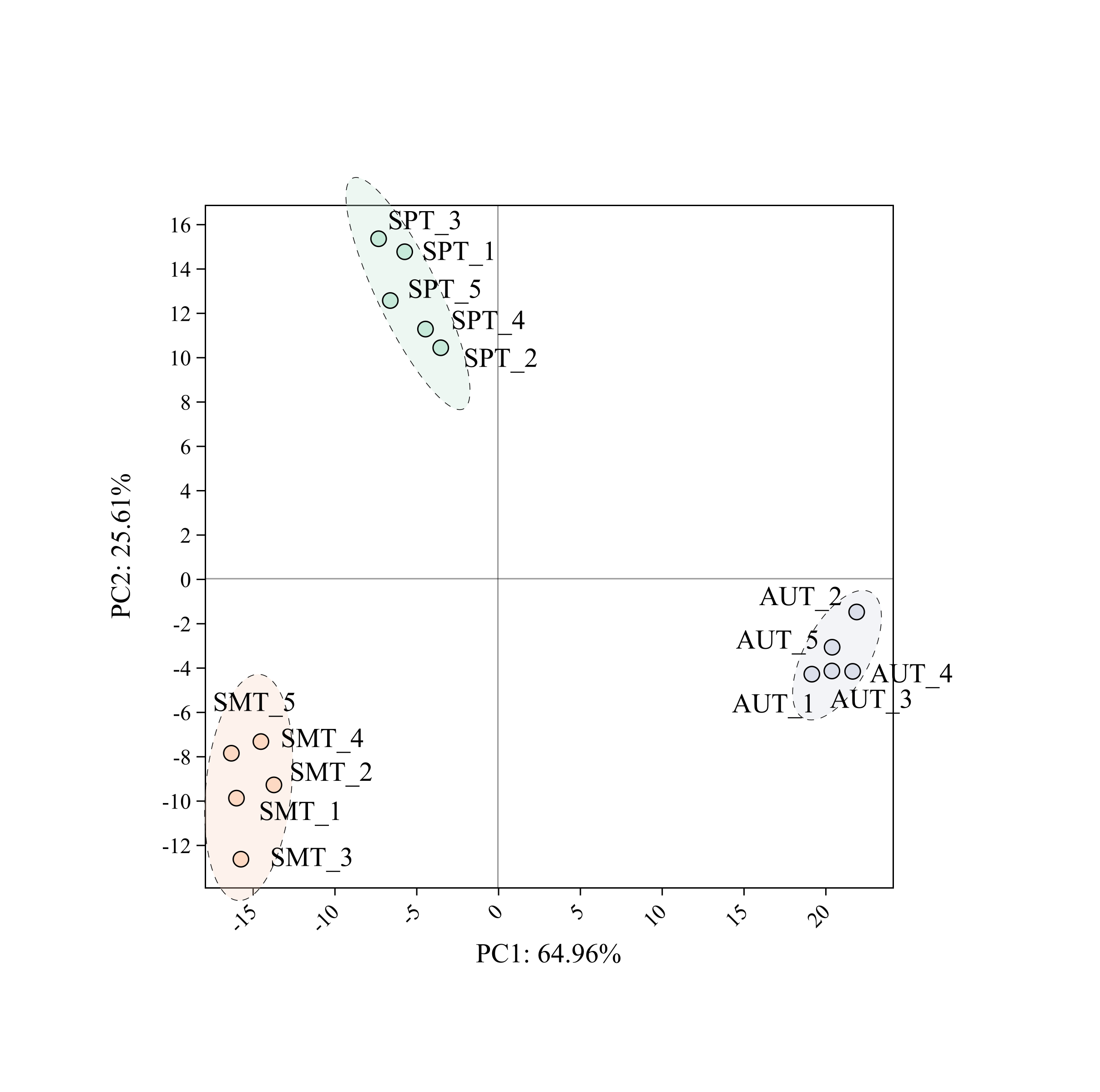

Supplement: Supplementary file 4 [file Image4.png]

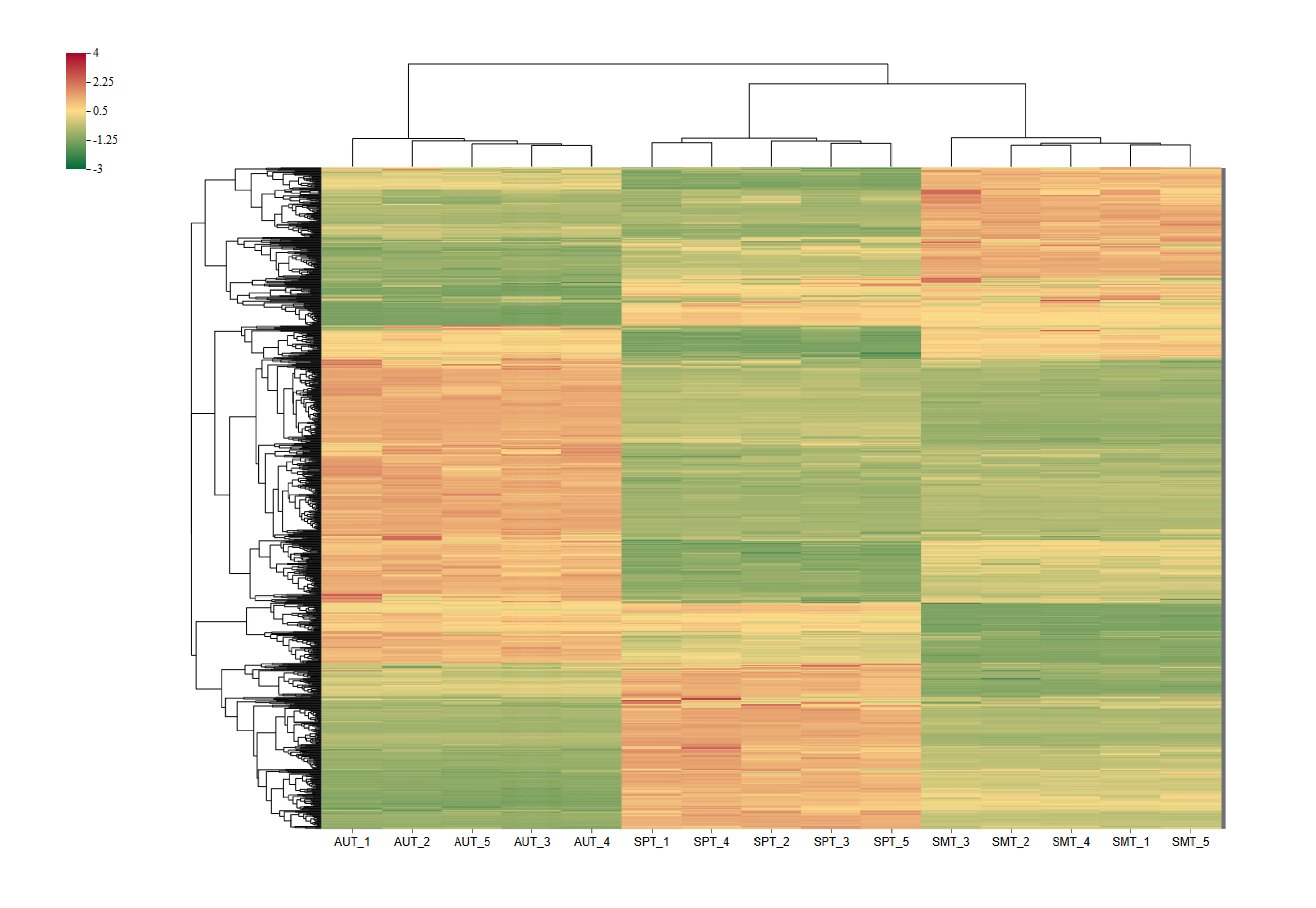

Supplement: Supplementary file 6 [file Image6.jpeg]
